# Characterizing Social Interaction in Tobacco-Oriented Social Networks: An Empirical Analysis

**DOI:** 10.1038/srep10060

**Published:** 2015-06-19

**Authors:** Yunji Liang, Xiaolong Zheng, Daniel Dajun Zeng, Xingshe Zhou, Scott James Leischow, Wingyan Chung

**Affiliations:** 1 School of Computer Science, Northwestern Polytechnical University, Xi’an, Shaanxi, China; 2 Department of Management Information Systems, University of Arizona, Tucson, Arizona, USA; 3 State Key Laboratory of Management and Control for Complex Systems, Institute of Automation, Chinese Academy of Sciences, Beijing, China; 4 College of Medicine, Mayo Clinic, Scottsdale, AZ, USA; 5 Department of Decision and Information Sciences, School of Business Administration, Stetson University, DeLand, FL, USA

## Abstract

Social media is becoming a new battlefield for tobacco “wars”. Evaluating the current situation is very crucial for the advocacy of tobacco control in the age of social media. To reveal the impact of tobacco-related user-generated content, this paper characterizes user interaction and social influence utilizing social network analysis and information theoretic approaches. Our empirical studies demonstrate that the exploding pro-tobacco content has long-lasting effects with more active users and broader influence, and reveal the shortage of social media resources in global tobacco control. It is found that the user interaction in the pro-tobacco group is more active, and user-generated content for tobacco promotion is more successful in obtaining user attention. Furthermore, we construct three tobacco-related social networks and investigate the topological patterns of these tobacco-related social networks. We find that the size of the pro-tobacco network overwhelms the others, which suggests a huge number of users are exposed to the pro-tobacco content. These results indicate that the gap between tobacco promotion and tobacco control is widening and tobacco control may be losing ground to tobacco promotion in social media.

Tobacco use is one of the biggest public health threats the world has ever faced, killing nearly 6,000,000 people each year and more than 5,000,000 of those deaths are caused directly by tobacco use^1^. Tobacco use is linked to the development of a number of serious illnesses including cancer, cardiovascular disease (CVD, such as hypertension, heart disease and stroke), and respiratory diseases^2^. Among the over 4,800 chemicals in tobacco, 61 of them are known to cause cancers including around 90% of lung cancer and also increase the risk of at least 13 other cancers encompassing cancers of the oral cavity, pharynx, larynx (voice box), esophagus, pancreas, kidney, bladder, and uterine cervix^3^,^4^,^5^. It is estimated that smoking causes nearly 10% of cardiovascular disease and is the second leading cause of CVD, after high blood pressure^6^. Chronic Obstructive Pulmonary Disease (COPD), a respiratory disease caused primarily by smoking, gradually makes it harder to breath. It is revealed that smoking accounts for 90% of COPD-death and increases the risk of developing COPD for youth tobacco users by slowing the growth and development of lungs. Also tobacco use is linked with mental health of human. Nicotine dependence can increase the risk of depression, and even suicide^7^,^8^. Beyond impacts on the individuals, tobacco use has far-reaching and long-lasting impacts on human being. The person-to-person spreading of smoking behavior through social ties^9^ and the transgenerational effect of nicotine^10^ pose great challenges for the tobacco control.

Although many smoke-free laws are enforced, it is difficult to fight the global tobacco epidemic in the age of social media via traditional tobacco control approaches. With more people embracing social media sites, particularly teenagers and youth adults, tobacco companies stand to benefit greatly from the marketing potential of social media. For example, cigarette promotion on Facebook and microblog Weibo^11^,^12^,^13^,^14^,^15^,^16^, pro-tobacco video clips on YouTube^17^,^18^,^19^,^20^,^21^,^22^ and mobile applications (‘ishisha’ and ‘Cigar Boss’) are established venues for tobacco promotion^23^,^24^. There is some evidence to suggest that exposure to the pro-tobacco content may turn potential tobacco users into regular tobacco users^25^. Meanwhile tobacco control communities are also utilizing social media to emphasize that tobacco use results in increased morbidity and mortality, and also to help smokers quit^26^,^27^. It is clear that social media is becoming a new battlefield for tobacco war between tobacco stakeholders and tobacco control community^28^,^29^,^30^.

With the escalation of tobacco “wars” in social media, the analysis of user interaction with tobacco-related content in social media is vitally important to properly assess the current situation, the potential risks and what kind of countermeasures to be taken. On one hand, the analysis of user interaction can facilitate the understanding of communications about tobacco products on nontraditional communication venues and reveal the tobacco product marketing in social media^31^. On the other hand, it provides significant insights for the tobacco control community to evaluate the program effectiveness, find the loopholes in tobacco control regulations, and further make reasonable decisions^32^.

However, few existing studies have conducted in-depth empirical studies on characterizing the user interaction in tobacco-oriented social media. In recent years, several case studies were conducted to uncover the hidden patterns of tobacco promotion activities and the prevalence of pro-tobacco video clips sponsored by the tobacco manufacturers in social media^11^,^14^,^18^,^20^,^33^,^34^, and some researchers also attempted to conduct online surveys to reveal the impact of favor on use of tobacco products^35^. Thus far, existing researches mainly rely on the user survey and the manual data extraction from questionnaires is time and labor-consuming. Furthermore, most existing researches are case studies and lack of quantification methods. With the rapid increase of tobacco-related content in social media, the existing methods for data collection and analysis cannot solve this problem well.

To fill this gap, in this paper, we characterize user interaction in tobacco communities based on large-scale social media information. Specifically, we construct three tobacco-related social networks and reveal the different patterns of user interaction in tobacco communities. The main contributions of this paper are two-fold: (1) To the best of our knowledge, it is the first time to collect large-scale tobacco-related data from online social media and uncover the tobacco interaction patterns in tobacco communities; (2) Based on the large-scale datasets, we characterize interaction patterns of collective activities in tobacco communities quantitatively and several significant results have been found. These results can help us to better understand the social interaction in tobacco communities and further make reasonable tobacco-control decisions.

## Results

We analyze the user interaction in tobacco communities from three perspectives including topological patterns of tobacco communities, statistical patterns of user interaction, and dynamical patterns.

### Topological Patterns of Tobacco Communities

**Figure 1:**
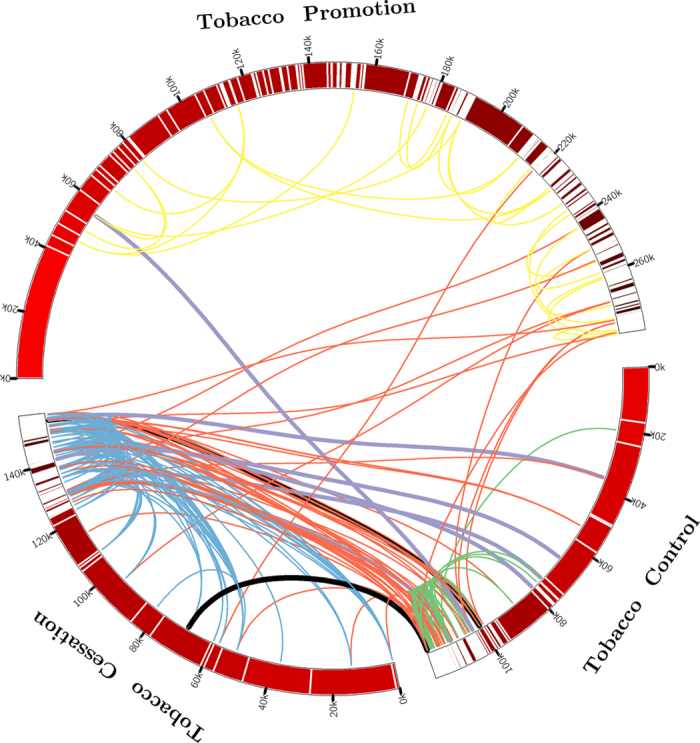
Comparison of interaction among tobacco communities. The width of sector indicates the volume of *page likes* for fan page. The color of sector implies the diversity of user interaction, namely interaction entropy, on fan pages. In each group, fan pages are sorted in descending order in term of interaction entropy.

Based on the user interaction records on Facebook, we construct three social networks, which are named as pro-tobacco network (PTN), anti-tobacco network (ATN) and quitting-tobacco network (QTN). For each network, the giant component is extracted and selected metrics are measured respectively. As shown in Table 1, the size of PTN is more than half of user volume in the whole dataset, which indicates many Facebook users are exposed to pro-tobacco content and interact with tobacco promotional content. For the average degree, QTN outperforms the other two networks, which means the connectivity of QTN is better. Meanwhile, the clustering coefficients of the three networks differ significantly as well. The lowest clustering coefficient of PTN (0.000023) reveals that the individuals interacting with the same pro-tobacco fan pages seldom know each other, and the user interaction is nearly random. By contrast, the clustering coefficient for QTN is significantly higher than a random graph constructed on the same vertex set. Percolation threshold *P*_*c*_ is the critical probability, in the vicinity of which the information percolates throughout the whole network. In term of percolation threshold *P*_*c*_, PTN and ATN have approximately the same threshold 0.5, which demonstrates that PTN and ATN perform same for the information dissemination. However, *P*_*c*_ is 0.25 for QTN, which implies QTN will facilitate the spread of information. In addition, the activities among tobacco communities are illustrated in Fig. 1.

### User Interaction in Tobacco Communities

#### Statistical Patterns of Page Features

**Figure 2:**
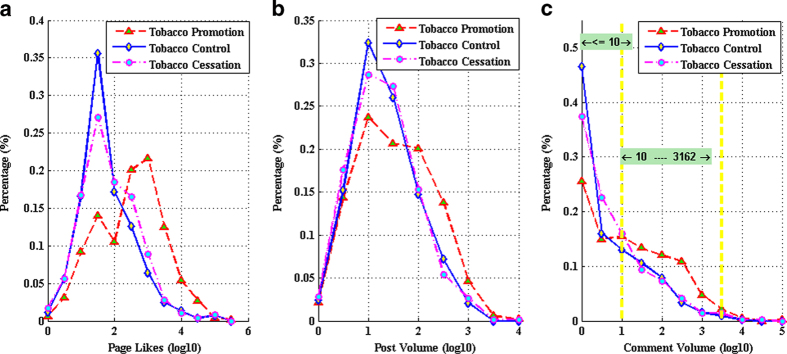
Statistical Patterns of Page Features in three tobacco communities .

To compare the user interaction in the three communities, we investigate the statistical patterns of page features including *page likes*, *post volume*, and *comment volume* respectively. The distribution of *page likes* in three groups is shown in Fig. 2a, where the horizontal axis represents the volume of *page likes* (measured by *log*_*10*_) and the percentage of fan pages with the given *page likes* is quantified on vertical axis. According to Fig. 2a, the curves of anti- and quitting- tobacco groups reach peaks at *log*_10_ (*pagelikes*) = 1.5, which means most of fan pages in those two groups have approximately 10^1.5^ ≈ 32 followers. By contrast, for the pro-tobacco group, it peaks at *log*_10_ (*pagelikes*) = 3 with a share of 21.6%, which demonstrates that 21.6% of pro-tobacco fan pages have approximately 10^3^ = 1000 followers. In addition, for both the anti-tobacco and quit-tobacco groups, the proportions decrease rapidly when the *log*_10_ (*pagelikes*) ≥ 2. Furthermore, we find that the pro-tobacco group overwhelms the other two groups when *log*_10_ (*pagelikes*) ≥ 3. This indicates that fan pages for tobacco promotion seem to be more effective to obtain user attention and user interaction.

Similarly, we analyzed the distribution of *post volume* and *comment volume* for each group. As shown in Fig. 2b, the three curves peak at *log*_10_ (*post*) = 1. However, when *log*_10_ (*post*) ≥ 2, the pro-tobacco group outnumbers the others. This reveals that many pro-tobacco pages are well maintained with more posts. As illustrated by Fig. 2c, it is observed that over 70% fan pages in anti-tobacco and quitting tobacco groups have less than 10 comments. On the contrary, it is about 56% for the pro-tobacco group. The high rate of pages with fewer comments indicates that more fan pages in anti-tobacco and quitting tobacco groups seem not successful in attracting user interaction. Especially, when *log*_10_ (*comment*) = 0, that is 46.5% for anti-tobacco, 37.4% for quitting tobacco and 25.6% for pro-tobacco. The high rate of pages with few comments demonstrates that many of those anti-tobacco fan pages and quitting tobacco fan pages do not benefit the campaign for tobacco control, and fail to help smokers to stop smoking with social support.

#### Uncertainty in User Interaction

**Figure 3:**
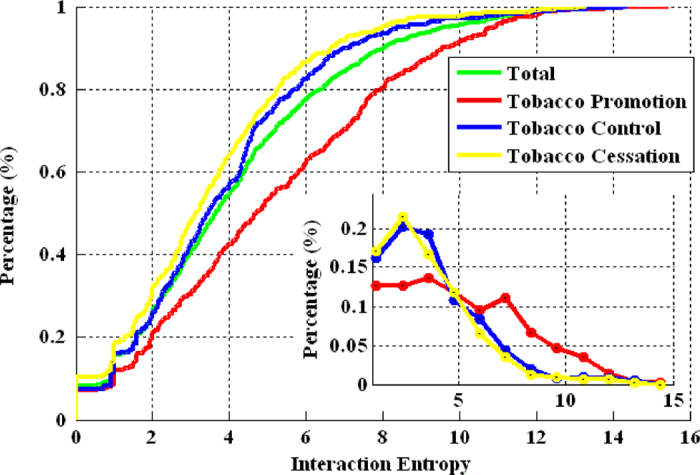
CDFs of interaction entropy for three tobacco communities. The growth rate of CDF for pro-tobacco community is the lowest. In the inset, when IE = 0, the percentage in pro-tobacco community is the smallest; while when *IE* ≥ *5*, the pro-tobacco community outperforms the other communities.

We measure whether the user interaction is active or not on the given fan pages utilizing information entropy^36^. In information theory, entropy is the average amount of information contained in each message and is best understood as a measure of uncertainty. In this paper, we use entropy to measure the uncertainty of user interaction in online tobacco-related communities. The users with higher entropy are regarded as active users. As shown in Fig. 3, the cumulative distribution functions (CDF) of anti-tobacco and quitting tobacco groups grow rapidly than that of global dataset; while the CDF of pro-tobacco group is significantly distinguished from others with lowest growth rate. It is illustrated that the user interaction in pro-tobacco group is more active. When *IE* ≤ *6*, the responding values for anti-tobacco group and quitting tobacco group are over 80%. However, it is approximately 60% for the pro-tobacco group. The gap in interaction entropy indicates that fan pages for tobacco promotion have succeeded in getting user attention with more users than the other fan pages.

The inset in Fig. 3 presents the distributions of interaction entropy for different groups. It is obvious that the percentages when *IE* ≤ *5* for both anti- and quitting- tobacco groups outstrip that of pro-tobacco group. Especially, compared with the over 20% for anti-tobacco group and more than 25% for quitting tobacco group when *IE* ≤ *0*, the percentage of pro-tobacco group is the smallest. On the contrast, when *IE > 5*, the pro-tobacco group outperforms the other two groups significantly. The emergence of fan pages with higher *IE* in pro-tobacco groups implies that fan pages for tobacco promotion are more effective for user interaction.

### Dynamic Patterns in Tobacco Communities

The popularity of social media such as Facebook and YouTube has raised the visibility of tobacco products and promotes tobacco use. Many users are exposed to the pro-tobacco user-generated content ranging from the product reviews to smoking fetish imagery to tobacco-related scenes. To reveal the impacts of those tobacco-related data to potential users, we measure the influence of tobacco-related fan pages in Facebook.

**Figure 4:**
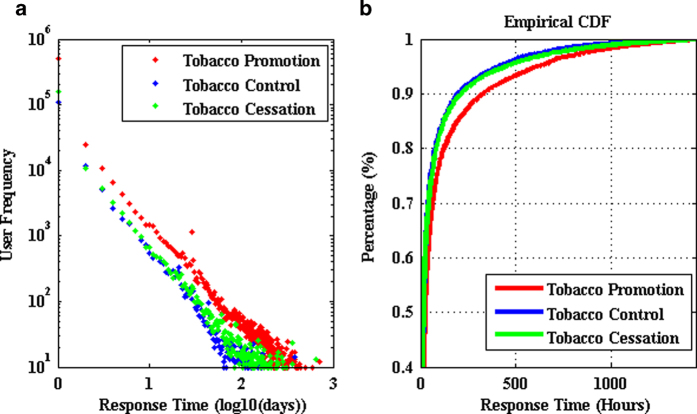
Distribution of response time in tobacco communities. 90% of user comments happened within less than 330 hours for pro-tobacco community; that is 197 hours for tobacco control and 213 hours for tobacco cessation respectively. This indicates that the posts in pro-tobacco community have long-lasting effects for online interaction.

With regard to social influence, we use the time series of comments on fan pages to reveal the influence of fan pages using the transfer entropy^37^. First, we need to analyze the response time of comments to set a reasonable bin width. We define time range as the time difference between timestamp of comments and initial time of post. Fig. 4a shows that the pro-tobacco group has a larger time range. As shown in Fig. 4b, 90% of user comments happened within 330 hours for the pro-tobacco group, while it is 197 hours for anti-tobacco group and 213 hours for quitting-tobacco group respectively. The longer time range for the pro-tobacco group indicates that user interaction in this group is more active and the content in pro-tobacco group has more long-lasting impact than that in other groups. On the other hand, for the whole dataset, 95% of comments happened within 163 hours, which indicates that a post will get most comments within 7 days. For the sake of simplicity, we choose the bin width as 7 days to calculate the transfer entropy.

The influence of fan pages is calculated respectively. Of the top 35 influential fan pages, 43% of them are conducting tobacco promotion. The pro-tobacco pages are devoted to tobacco campaigns with many strategies. *‘AnimalsSmokingDurrys’* and *‘GirlsSmoking’* are examples of using fetish imagery (images of young men and women smoking, smoking sexual fetish scenarios, smoking animals or cartoon characters etc.) to promote smoking as cool, fashion or fun. Online tobacco shops such as ‘*smokefreeonline*’ and ‘*hookah-shisha*’ and tobacco retails (‘*mrhookah*’ and ‘*bnbtobacco*’) create fan pages with embedded URLs of online tobacco shops, which raises the visibility of tobacco products and makes it more convenient for the potential buyers to access those websites for potential online tobacco purchase activities. Meanwhile, fan pages named after tobacco brands such as ‘*EcoDumas.lt*’ and ‘*espinosacigars*’ are established for tobacco brand campaign. On the other hand, the social media is utilized for tobacco control and tobacco cessation as well. Many regional organizations such as ‘*TobaccoFreeFlorida*’ and ‘*TobaccoFreeCA*’ were created for tobacco free campaigns. Furthermore, some tobacco cessation services are provided to help smokers to quit smoking. ‘*BecomeAnEX*’ is a very famous community to help the smokers by facts, therapy and experience sharing.

### Classification of User Interaction

In this paper, we present how to characterize the user interaction in tobacco-related communities using interaction entropy and transfer entropy. The metrics presented above could be utilized for the classification of user interaction. We classify user interaction into following 4 types: High TE High IE (*HH*), High TE Low IE (*HL*), Low TE High IE (*LH*) and Low TE Low IE (*LL*).

For *HH* pages, the administrators are very influential and user interaction on these pages is very active. For *HL* pages, it means the user interaction is very low but the influence of the page’s administrator is significant. On *LH* pages, although the administrators of pages are not very influential, the user interaction of these pages is pretty active. Intuitively, the administrators of *LL* pages are not active with low influence on the followers.

**Figure 5:**
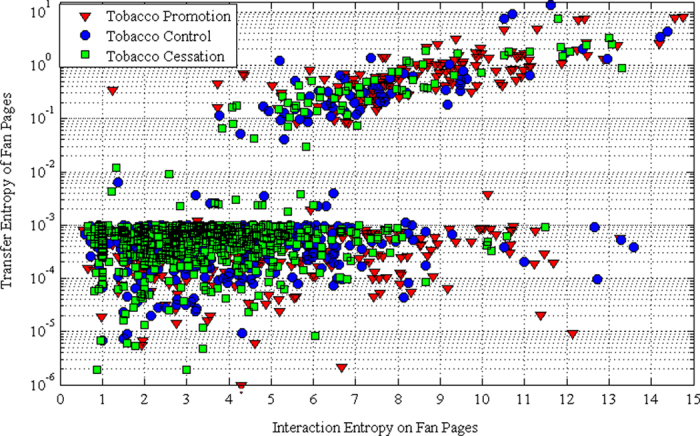
Page classification according to interaction entropy (IE) and transfer entropy (TE).

**Figure 6:**
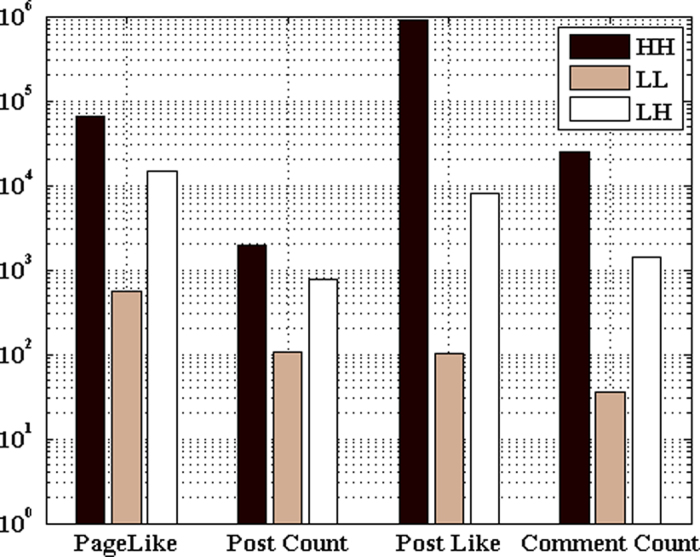
Comparison of page features in different groups.

As presented in Fig. 5, we find that *LL* pages in the dataset are overwhelming, which implies that although many tobacco-related fan pages are created on Facebook, but most of them are inactive and less influential with low interaction entropy and low transfer entropy. *LL* pages may result from the following reasons: (1) abandoned pages: the administrators of fan pages did not maintain the pages very well, finally resulting in the page abandoned or deleted. As shown in Fig. 6, the volume of posts on *LL* pages is very low. The bad maintenance could be presented in many different forms. Some pages are created purposely to gain more page likes without any interesting post updates. Therefore, they are only active in very short time; (2) newly-born pages: pages are created very close to the interval of data collection. For the newly-born pages, only a few posts are released with few comments and page likes. Therefore, the newly-born pages are identified as *LL* pages.

It is very intuitive that *HL* pages are very rare. The definition of *HL* pages is contradicting with the common sense that the influential users are more likely to impact or change peer behavior^38^,^39^. When the user interaction is very low, it indicates the page is less attractive for the Facebook users. Therefore, it is difficult to gain high influence on pages with low interaction entropy. While for *HH* pages, it is very convenient to launch commercial campaigns. On one hand, the high user interaction makes a huge number of users exposed to the user-generated content, finally raising the visibility of commercial products. On the other hand, the high influence of the administrators boosts the information dissemination beyond the local community. As presented in Fig. 5, *HH* pages for tobacco promotion are overwhelming. For example, ‘*AnimalsSmokingDurrys*’ promotes smoking is fun with smoking animals. Although many regional tobacco control campaigns are launched such as ‘*TobaccoFreeCA*’ and ‘*smoke.free.mich*’, the tobacco control community is losing the ground in the tobacco war on social media with fewer *HH* pages for tobacco control.

Distinct from *HH* pages, the administrators of *LH* pages are less influential. To reveal the differences between *HH* and *LH* pages, we investigate the comment patterns and post sources. With regard to post sources, we define *R* as the rate of volume of posts released by the administrator and total post volume on the given page. For the *LH* pages, *R*_*LH*_ = 0.2656, while it is *R*_*HH*_ = 0.7151 for *HH* pages. This implies that it is the fans instead of administrators of *LH* pages who maintain the pages. More importantly, it indicates the promotional strategies of two kinds of fan pages. For *HH* pages, it is the administrators who maintain the pages. The strategy is named as the central mode. While for the *LH* pages, it is the potential users instead of the administrators who play important roles in the posting updates. This is named as the crowdsourcing mode. On the other hand, the difference of average comment volume per post (*CVP*) of the two kinds of pages is significant (*CVP*_*HH*_ = 6.2281, *CVP*_*LH*_ = 1.6730). The lower *CVP* for *LH* pages results in the low transfer entropy.

## Discussion

Our empirical studies demonstrate that the exploding pro-tobacco content has long-lasting impact with more active users and more powerful influence, and reveal the shortage of social media resources in global tobacco control. With regard to user interaction and social influence, we find that user interaction in the pro-tobacco group is more active, and fan pages for tobacco promotion are more successful in obtaining more user attention. These results indicate that the gap between tobacco promotion and tobacco control is widening and tobacco control is losing ground to tobacco promotion in social media. However, the work in this paper is subject to some limitations.

First, to reveal the differences of tobacco-related networks, the coverage of dataset is crucially important. To overcome this problem, we build a set of tobacco-related keywords with the integration of three heterogeneous data sources including tobacco brands, synthetic tobacco-terms and existing knowledge to maximize the coverage of dataset. For tobacco brands, we integrate tobacco brands from online tobacco shops and tobacco review websites to cover as many as tobacco brands in our dataset. For synthetic tobacco-terms, we gathered a large number of tobacco-related words, which cover both tobacco promotion and tobacco control activities as well. In addition, varieties of tobacco products (such as cigar, snuff, and hookah) and even tobacco accessories such as pipe are included. Although all those methods are not enough to ensure the completeness of the keyword set, it does benefit to broaden the coverage of the keyword set. In addition, our method is scalable with the introduction of new keywords. This feature makes the dataset reasonable to reveal the user interaction on tobacco-related communities.

Second, we employ the interaction entropy and transfer entropy to reveal the diversity and influence of user interaction respectively. For the diversity of user interaction, many kinds of user activities such as *post like*, *comment* and *sharing* are included. While, for the transfer entropy, only comment records are counted. On one hand, the timestamp of *post like* is not available. The deficiency of the *post like* records makes it unfeasible to reveal the dynamic patterns of social networks. On the other hand, legitimate companies are being flooded with fake likes on Facebook^40^,^41^. The fake likes lead to pages overwhelmed by useless followers and render the distorted metrics. For example, likefake (http://likefake.com) is a simple tool to boost the popularity with fake likes. To address this problem, we only utilize the comment records to measure the influence of fan pages. The comments on posts are assumed as interaction activities from valid users instead of fake, abusive accounts. By eliminating fake likes, we will gain better insight into how many people are truly active. Furthermore, we will gain more accurate views as to their influence in tobacco communities.

Although there are some limitations in this paper, this work could contribute to many applications. According to Food and Drug Administration’s research priorities, the analysis of user interaction in tobacco-related social media helps us to understand the extent of tobacco production discussions and communications in non-traditional venues^31^. For the tobacco control communities, user interaction could be employed to measure the effectiveness of tobacco control campaigns on social media, demonstrate the most effective messages regarding regulatory authority over tobacco products and reveal what are the best communication avenues to convey messages to the public^31^. On the other hand, tobacco companies stand to benefit greatly from the marketing potential of social media, without themselves being at significant risk of being implicated in violating any laws^11^. It is crucial to remove tobacco-related sale campaigns on social media from a huge number of user-generated content automatically. The analysis of user interaction reveals the user interaction patterns in the sale campaigns, which makes it possible to differentiate the sale campaigns from other tobacco-related content. Meanwhile, the research progress in other field such as the research about obesity^42^,^43^,^44^ could be applied to tobacco-related research to reveal the spreading and evolution of tobacco-related social works.

## Methods

### Data Collection

In this paper, we mainly focus on the user interaction on tobacco-related Facebook fan pages. Firstly we construct a set of tobacco-related keywords including tobacco brands, synthetic tobacco-terms, and existing knowledge to find the tobacco-related fan pages. Due to the diversity of tobacco brands, we integrate tobacco brands from four kinds of sources including the official tobacco brand list, tobacco-brand related wiki web pages, tobacco review web sites and online tobacco shops. Totally, we obtained 186 tobacco brands and counted the number of times that brand occurs in those data sources respectively. Finally, we chose the representative brands according to the average of frequency of occurrence. Totally, we got 70 popular brands. For the synthetic keywords, we synthesize tobacco-related words with different roots (as shown in Table 2). In addition, some famous existing tobacco-related fan pages such as ‘quitnet’, ‘BecomeAnEX’ and ‘VchangeU’ and tobacco-related products including ‘beedi’, ‘cigar’, ‘snuff’, ‘hookah’, and ‘pipe smoking’ are supplemented as well. Fan pages regarding the given keywords are retrieved through RestFB^45^, which is a flexible and open source tool to access the Facebook Graph APIs^46^.

All retrieved fan pages are classified manually into 4 types according to page profiles and content of fan pages. Specifically, we first classified the retrieved results into 4 types by two coders manually: (0: unrelated to tobacco; 1: tobacco promotion; 2: tobacco control; 3: tobacco cessation) and then utilize the Cohen’s Kappa coefficient^47^, with a high coding reliability (K = 0.9519), to measure the similarity of classification results by these two coders. When there is no agreement between the first two coders, we let the third coder code the fan pages. If the third coder disagreed with each of the first two coders, the fan pages are excluded. Totally, we got 2149 tobacco-related fan pages (708 for tobacco promotion, 684 for tobacco control and 757 for tobacco cessation).

For the tobacco-related fan pages, features of fan pages including the volume of *page like*, *post volume*, *comment volume* and *response time* are collected. In addition, the user interaction activities are gathered as well. Here, we define one interaction between *user* A and *user* B when *user* A comments (likes or shares) a post posted by *user* B. The dataset was collected by our implemented tobacco surveillance system in the spring of 2013^12^. According to the user interaction records, we construct an undirected weighted interaction graph ***G*** = {*V, E*}, where *V* = {*v*_*1*_*,v*_*2*_*,…v*_*i*_*,…v*_*n*_} indicates users who interact with posts; *E* = {*e*_*1*_*,e*_*2*_*,…,e*_*i*_*,…e*_*m*_} is the set of edges which connect *v*_*i*_ and *v*_*j*_ with interaction frequency *w*_*ij*_. According to the labeling of fan pages, we could construct three interaction graphs respectively. The sizes of three interaction graphs are presented in Table 3.

### Measuring Network Topology

To compare the three tobacco-related communities, we investigate the connectivity, transitivity and robustness respectively using the metrics presented in Table 4. Degree distribution reveals the connectivity of network. The first moment *<k>* indicates the mean degree of the whole network. The degree *k* of node *i* is the count of edges incident with the node *i*, and *P(k)* is defined to measure the fraction of vertices in the network that have degree *k*^48^. To measure the transitivity of one social network, clustering coefficient *C* is introduced to quantify the likelihood that two neighbors of a node are associated with themselves. The modularity reveals the extent to which nodes cluster into community groups. Suppose that nodes in the network are partitioned into communities, where *c*_*i*_ records the community membership of node *n*_*i*_. The modularity of the partitioning is presented in Table 4, where *m* is the number of edges, *A* is the adjacency matrix, *k*_*i*_ and *k*_*j*_ represent the degrees of node *i* and *j*, and *s*_*i*_*s*_*j*_ = 1 if node *i* and *j* belong to the same community and −1 otherwise^49^. Robustness (also resilience) is defined as the ability of system to maintain its connectivity properties after random deletion of a fraction of its nodes and edges. Generally this problem is analytically treated by using percolation theory^50^,^51^ to find the critical point, symbolized by *P*_*c*_. The value of critical point can be calculated precisely for some lattices. For the Bethe lattice^52^, *P*_*c*_ is precisely computable^53^. As shown in Table 4, *P*_*c*_ for Bethe lattice is dominated by *z*, where *z* is the number of immediate neighbors of a vertex. According to^54^, *z* could be approximated to the average degree of social network.

### Quantifying User Interaction

To quantify whether the user interaction is active or not, we employ the Interaction Entropy (IE)^36^, which can be utilized to measure the diversity of user interaction on fan pages. The more active is the user interaction on fan pages, the higher is the interaction entropy. Specifically, for each fan page, we collected the interaction history of users. If *user i* comments (likes or shares) on a post shared on *page S*, it is regarded as interaction happened between *user i* and *page S*. We collected the interaction activities and the interaction frequency among users using a triple, symbolized by *I* = *(S, u*_*i*_*, w*_*i*_), where *u*_*i*_ and *S* are the unique IDs of *user i* and *page S* respectively, and *w*_*i*_ indicates the interaction frequency between *user i* and *page S*. Therefore, the interaction entropy of *page S* could be measured according to Equation 1, where *p*_*i*_ represents the probability of interaction between *user i* and *page S*, and *n* indicates the volume of users who interact with *page S*. For the user interaction on fan pages, it reveals the diversity of user interaction. When the interaction entropy is high, it means more users interact with *page S*.


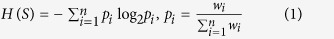


### Identifying Social Influence

In this paper, transfer entropy is utilized to reveal the fine-grained interaction between peers and explore the evolution of network from a macroscopic view. First, we regard the chronological user activities as a stochastic process. Then for each user (denoted as *X*) in online social networks, we record the history of interaction activities into a stochastic process *X*_*t*_. The dynamics of user *X*, symbolized by *H(X)*, is defined as Equation 2, where *p*(*x*) is the probability of *H*(*X* = *x*_*i*_). Consider two random users *X* and *Y*, the joint entropy *H(X,Y)* and conditional entropy *H(X|Y)* for *X* and *Y* are defined, respectively, as Equation 3 and 4, where *p*(*x,y*) and p(*x*|*y*) are the join probability and conditional probability respectively. We now turn to stochastic processes. For two users *X* and *Y*, their activities are represented by two stochastic processes *X*_*t*_ and *Y*_*t*_. The reduction of uncertainty about *X*_*t+1*_ due to the information of the past state 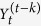 of *Y*, represented by 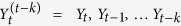, in addition to the information of the past 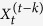 states of *X*, represented by 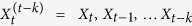, is measured by the transfer entropy from *Y* to *X*, defined as^55^


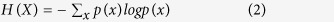



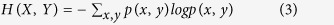



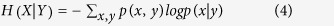



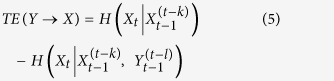


In Equation 5, the first term represents the uncertainty about *X*_*t*_ given *X’*s history only. The second term represents the uncertainty when *Y’*s history is given as well. Similar definition holds for 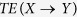. For the sake of simplicity, we take *l* = *k* henceforth. The transfer entropy between two stochastic processes is asymmetric and is characterized as the reduction of uncertainty in one process due to the knowledge of the other process^56^,^57^. In^58^, transfer entropy is introduced to measure the peer-to-peer influence among social media users. Similarly, we quantified the peer influence in tobacco-related social networks. Then we investigated the influence of a given fan page from the perspective of network. Given a network ***G ***=*** ***{*V*,*E*}, where *V* = {*v*_*1*_*,v*_*2*_*,…v*_*i*_*,…v*_*n*_} indicates users who interact with posts; *E* = {*e*_*1*_*,e*_*2*_*,…,e*_*i*_*,…e*_*m*_} is the set of edges which connect *v*_*i*_ and *v*_*j*_, the influence of node *v*_*i*_ in ***G*** at given timestamp *t* is defined as Equation 6, where *C(S)* indicates the set of nodes (*v*_*j*_) who interacted with node *v*_*i*_


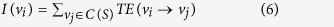


With the evolution of social network, the social influence of node *v*_*i*_ is time varying. Therefore, we could measure the evolution of social influence by time series of user interaction activities according to Equation 6. In this paper, we utilize releasing time of posts and the timestamps of comments to measure transfer entropy. Given *page S*, we build the stochastic process of page *S* according to timestamps of posts. While, for each user *v*_*j*_ who comments the posts on *page S,* we extract the time series of comments as stochastic process respectively. Then we calculate the evolution of influence of *page S* according to Equation 6.

## Additional Information

**How to cite this article**: Liang, Y. *et al*. Characterizing Social Interaction in Tobacco-Oriented Social Networks: An Empirical Analysis. *Sci. Rep.* **5**, 10060; doi: 10.1038/srep10060 (2015).

## Figures and Tables

**Table 1:**
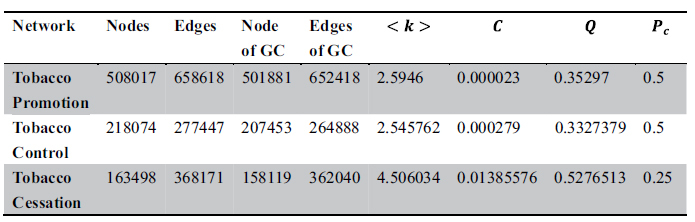
Comparison of network metrics on tobacco-oriented social networks.

**Table 2:**
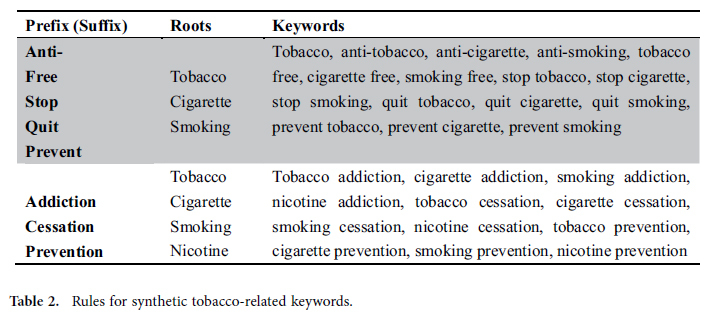
Rules for synthetic tobacco-related keywords.

**Table 3:**
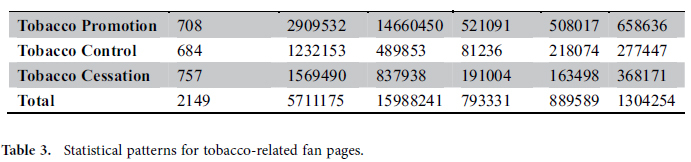
Statistical patterns for tobacco-related fan pages.

**Table 4:**
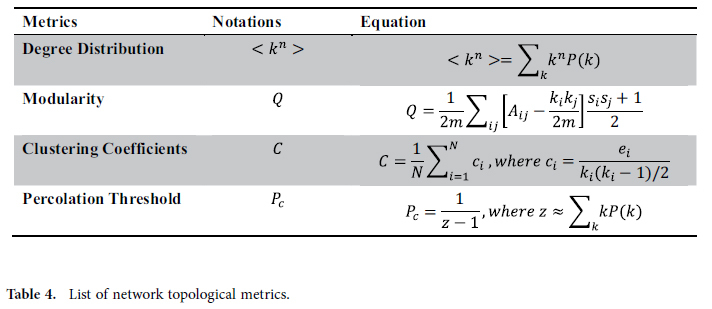
List of network topological metrics.
